# Regulatory impact of intermittent fasting on autophagy in high fat diet induced structural and cognitive brain deteriorations in rats

**DOI:** 10.1038/s41598-026-52334-9

**Published:** 2026-05-25

**Authors:** Mohamed Aref, Shimaa Hadhod, Nievin Ahmed Mahran, Haifa A. Alqahtani, Mohamed H. A. Gadelmawla, Mustafa Abed Elsalam Elzohier, Sara Ali Kandeel, Amira F. A. Ahmed, Amira Hamed, Sahar M. El-Sayed, Eman H. Elsheikh, Nanees F. El-Malkey

**Affiliations:** 1 Department of Anatomy and Embryology, Faculty of Veterinary Medicine, Zagazig University, El-Sharkia, 44519 Egypt; 2 Medical Physiology Department, Faculty of Medicine, Zagazig University, El-Sharkia, Egypt; 3 Biochemistry Department, Faculty of Dentistry, Sinai University – Kantara, Ismailia, Egypt; 4 Department of Biology, College of Science, Imam Abdulrahman Bin Faisal University, 31441 Dammam, Saudi Arabia; 5 Histology Department, Faculty of Dentistry, Sinai University, Kantara Campus, Ismailia, Egypt; 6 Department of Neurosurgery, Faculty of Medicine, Zagazig University, El-Sharkia, Egypt; 7 Medical Histology and Cell Biology Department, Faculty of Medicine, Zagazig University, El-Sharkia, Egypt; 8 Human Anatomy and Embryology, Faculty of Medicine, Zagazig University, El-Sharkia, Egypt; 9 Comparative Anatomy Department, Faculty of Science, Zagazig University, El-Sharkia, Egypt

**Keywords:** Intermittent fasting, Obesity research, High fat diet, Cognitive brain disorders, Diseases, Neurology, Neuroscience

## Abstract

Obesity-induced cognitive decline has been linked to alterations in brain autophagy. However, research concerning the high-fat diet (HFD) impacts on the brain still lacks evidence, and results are controversial. Intermittent fasting (IF) may lead to increased neurogenesis levels in the hippocampus in neurodegenerative diseases; however, the involved molecular mechanisms are not well understood. The current work aimed to evaluate the neuroprotective effect of IF against obese rat model-related cognitive disorders that disrupted brain autophagy. 24 male rats were allocated to control, fasting lean group, obese (HFD-fed), and obese fasting groups; behavioral tests, biochemical assays, and molecular analyses (inflammatory markers, BDNF, and autophagy-related genes) were conducted to assess cognitive function and underlying mechanisms. Our findings suggest that IF intervention significantly attenuated HFD-induced cognitive impairment and neuroinflammation, increased BDNF levels, improved histological alterations, decreased Beclin-1 and p62 immunohistochemical expression, and upregulated LC3 and ATG5 mRNA expression. IF can prevent HFD-induced cognitive disorders that could be mediated by the cerebral cortex and hippocampal autophagy dysfunction, emphasizing the importance of the autophagy pathway to normal neuronal functions. These results suggested that IF protected the neural system from HFD-induced inflammation and oxidative stress in obese rats and is essential for neuronal survival via modulation of autophagy function in rats.

## Introduction

Over the past few years, there has been a noticeable increase in dietary fat intake, and since 1980, the prevalence of obesity has doubled worldwide^1^. Numerous indicators demonstrated the detrimental effects of obesity and the diseases it is linked to, including cardiovascular disease, Alzheimer’s disease (AD), psychiatric disorders, hyperlipidemia, and hypertension^1–3^. According to epidemiological research, those who consume a lot of saturated fats over an extended period run a significant risk of developing dementia and cognitive impairment^4^. Nevertheless, the mechanisms underlying how consuming a high-fat diet (HFD) affects cognition remain inadequately understood^3^.

Several pathophysiological mechanisms have been shown to contribute to the cognitive impairment caused by obesity^5^, such as decreased brain vascular integrity and compromised blood–brain barrier which is followed by activation of neuroinflammation and neurodegeneration^6^. In addition, research on animal models of obesity revealed that long-term high-fat diets (HFDs) cause increased leakage of plasma-derived IgG into the perivascular region of the hippocampus and decreased microvascular density in the hippocampus, which impairs hippocampal-dependent cognitive function^7^.

Furthermore, autophagy is a process for cell survival that is necessary for maintaining cellular homeostasis. It makes it possible to recycle old cellular components and eliminate damaged organelles and protein aggregates^8^. It’s interesting to note that autophagy plays a role in eliminating neuronal tau and amyloid β clumps^9^, that are known to accumulate by defective autophagy responses, exacerbating cognitive dysfunction in several neurodegenerative diseases^9,10^. Therefore, autophagy activation has a critical neuroprotective role in brain damage and neurodegenerative diseases^11,12^. However, research concerning the HFD impacts on the brain still lacks evidence^13^. A steady number of research state that altered autophagy has been detected in obese humans and animal models of obesity, despite the fact that assessing autophagy in obesity is difficult and the results described in the literature are sometimes contentious^13,14^, there is disrupted autophagy in HFD mice’ brain^13^.

Intermittent fasting (IF), in which people alternate periods of regular food consumption with extended periods of little to no meal consumption^15^, showed beneficial results against obesity and its related co-morbidities, in addition to its numerous profits in general health like keeping a normal range of glucose level in the blood, reducing insulin and inflammation^16,17^. Moreover, earlier research looked into its potential neuroprotective advantages. According to reports, IF may raise the hippocampal neurogenesis levels in mouse models of neurodegenerative disorders and acute brain injuries like stroke^18^ and neurodegenerative diseases in rodent models^19,20^. As pharmacotherapy, lifestyle changes like diet and exercise have been proposed as having a role in the management and prevention of diseases marked by cognitive impairments, such as dementia, for which medication has demonstrated minimal benefits^21^. In addition, emerging evidence also implicated IF-induced modulation of autophagy in overweight/obese participants^22^ and in cancer patients^23^. Nevertheless, little is known about the molecular processes underlying IF-induced neurogenesis and brain autophagy regulation^24^.

Although prior research has independently addressed the cognitive, inflammatory, or autophagic consequences of high-fat diet exposure, there remains a lack of integrative studies evaluating these factors concurrently. To our knowledge, no previous study has examined the interplay between neuroinflammation, autophagy dysregulation, and neuroplasticity in the context of HFD-induced cognitive decline, particularly in response to IF. Thus, the goal of the current study to addresses this gap by adopting a multi-level approach that connects behavioral, molecular, and histological outcomes in a trial to offer new insights into the pathophysiological mechanisms of IF in an obese rat model.

## Material and methods

### Animals

Twenty-four adult (2 months old) male Sprague Dawley rats weighing (220–260 g), were brought from the animal house of Faculty of Medicine, Zagazig, University, Egypt. Male rats were selected to avoid estrous-related hormonal changes affecting brain physiology.

Animals were held in plastic cages (3/cage) within antiseptic conditions at a temperature of 25 ± 2 °C, and a normal light/dark cycle of 12 h with free access to food and water for two-weeks housing period to accommodate for the lab environment.

The current protocol has been revised and permitted by Zagazig University- Institutional Animal Care and Use Committee (ZU-IACUC) according to the U.K. Animals (Scientific Procedures)^25^ and the National Research Council’s Guide for the Care and Use of Laboratory Animals^26^ and in compliance with the ARRIVE guidelines, The approval number: ZU-IACUC/3/F/168/2024.

### Experimental design

Four equal groups were randomly selected from among the experimental animals (n = 6/each) as follows:

- Control group (Control): Received a standard chow diet throughout the experiment.
- Fasting lean group (FL): Fed a standard chow diet on four nonconsecutive days per week, alternating with 24-h fasting on the remaining three days, following the protocol of^27^
- Obese group (Obese): Fed a high-fat diet (HFD) for 2 months to induce obesity. The HFD consisted of 58.3% fat, 20.2% protein, and 21.5% carbohydrates, providing 5.40 kcal/g, as described by^28^ and prepared by the Faculty of Agriculture, Zagazig University, Egypt.
- Fasting obese group (FO): Fed the same HFD on four nonconsecutive days per week, alternating with 24-h fasting on the other three days, with fasting hours started at 6pm to 6pm next day for the entire 2-month period.

Animals were weighed by electronic balance (Germany) weekly, and body mass index (BMI) for all animals was calculated at the end of the study to ensure the development of obesity^29^.

**Fig. 1:**
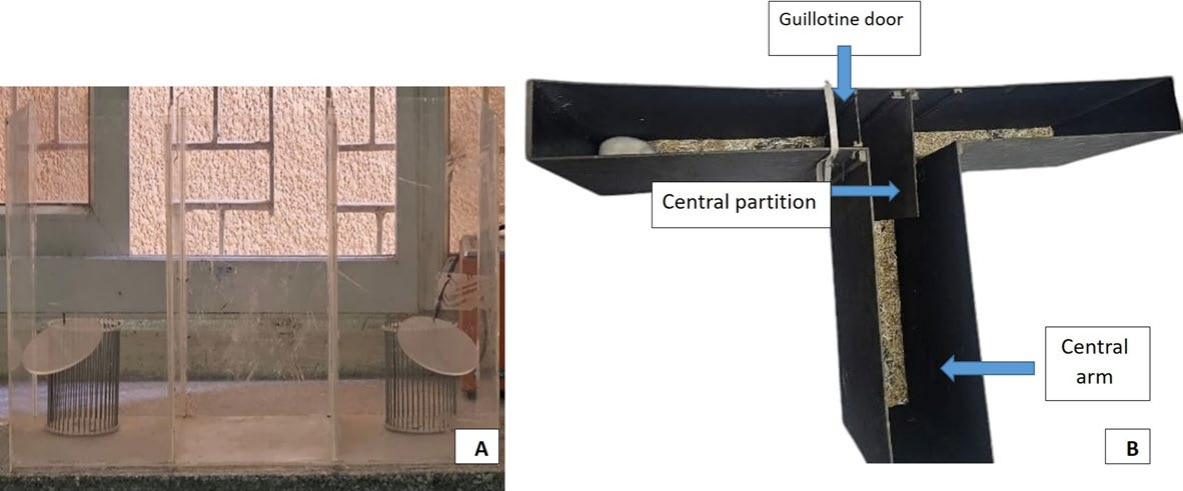
(**A**): Apparatus for anxiety testing, (**B**): Modified T-Maze.

At the end of the study period, the following examinations were done as *behavioral tests*:

A. A hand-built rectangular acrylic box with three equal-sized rooms divided by retractable doors is used for the anxiety testing "Crawley’s Sociability Test" as follows:

Two similar empty circular wire cups were placed vertically in the center of each side chamber, with the test rat in the center, to confine the unidentified rats (Fig. 1A). Social memory and novelty, as well as social attachment and motivation, can be evaluated thanks to the experimental design of this test. The test rat is allowed to spend time in any of the three box compartments during the experimental session, which includes indirect interaction with one or two unfamiliar rats.

The test comprised three sittings (10 min each) as sitting 1. “Habituation”: the test rat was positioned in the center compartment while the exterior doors were shut. There were the same empty wire cups on both sides, sitting 2. “Social Affiliation”: in this experiment, a control, the unknown rat was assigned the name “Stranger 1” and kept in the wire cup in one of the side chambers. The “test” rat was given unrestricted access to all three chambers by opening the doors between them, and Sitting 3 “Social Novelty” component at which a second control unfamiliar rat, "Stranger 2," was positioned within the wire cup in the opposite side chamber, video camera was used to record and monitor the observations.

To ensure proper decontamination and avoid bias and olfactory indicators, 70% ethanol was used to disinfect every chamber after every trial. The amount of time the test rat spent in each compartment overall was estimated after the experiments^30^.

B. *Depressive-like Behavior Test (Forced Swim Test (FST))* involves watching a single rat’s behavior for five min in a glass cylinder filled with water at 25 °C. An indicator of depressive behavior was the immobility time. Each rat’s swimming, climbing, and immobility times were then computed^31^.

C. *Memory testing* using "A manually run Modified T-Maze" (Fig. 1B) as all of the guillotine doors were raised, and the maze was arranged with the central divider in place. Each run began with the rat at the bottom of the T, and it was given the freedom to select a goal arm. The door was softly slid down to keep the rat in the selected arm for 30 s. After that, it was carefully taken out and put back in the cage for a 10-min break between trials. Instead of facing the goal arms, the rat was positioned at the start area and given the option to select one of the two open goal arms. Each test took one to 2 min. In contrast to the previous run, alternation was defined as the rat entering the opposite arm.

Over two days, each rat underwent one sample trial and five choice runs daily, for a total of 12 trials and 10 possible alternations. The following formula was used to determine each animal’s proportion of the right option (alternation): The number of right answers (alternations) divided by the total number of potential answers × 100^32^.

Then after overnight fasting, the animals were decapitated under anesthetic overdose (intraperitoneal overdose of sodium pentobarbital (150 mg/kg)) to induce deep anesthesia followed by euthanasia to ensure rapid loss of consciousness and minimize animal distress. This approach is consistent with the principles outlined in the AVMA Guidelines for the Euthanasia of Animals^33^ and blood was collected to estimate serum IL-1β and Tumor Necrosis Factor-α (TNF-α), as described by^34^.

### Macromorphological examination of the brain and hippocampus

For brain dissection, the heads of the rats were opened under aseptic conditions. The skull was carefully cut at the level of the occipital, parietal, and frontal bones, which form the cranial roof, using curved scissors to expose the cerebral cortex for photography. Subsequently, the entire brain was gently removed from the cranial base to allow access to the hippocampus.

Each brain was divided into two hemispheres: One half was allocated for histopathological and immunohistochemical (IHC) examination. The other half was further divided to perform both real-time polymerase chain reaction (RT-PCR) and ELISA analyses from the same animal.

### Preparation of Tissue Homogenate

Each experimental rat’s hippocampus and frontal cortex were homogenized using a hand-held homogenizer (Omni International, Kennesaw, GA, USA) in an ice-cold medium of 1.15% KCl (pH 7.4) prepared at a ratio of 10% (w/v) mixture (0.1 g of tissue per 1 mL of solution). Centrifugation at "1000 × gravitational force for 15 min" produced the supernatant fluid, which was then utilized with rat ELISA kits (Abcam Limited; USA) to further analyze brain-derived neurotrophic factor (BDNF), TNF-α, IL-1β, malondialdehyde (MDA), and superoxide dismutase (SOD).

### Histopathological technique

Following a 24-h fixation period in 10% neutral buffered formalin, the cerebral cortex and hippocampal specimens were dehydrated in graded ethanol, cleared in xylene, and embedded in paraffin. They were then sectioned into tissue sections that were 5 μm thick, stained with hematoxylin and eosin (H&E), and examined under a microscope for any histopathological changes^35,36^.

All section photos were taken using a Swift microscope associated with Swift digital camera. we used a semiquantitative scoring system (0–3) to evaluate characteristic neuropathological features, including: Pyknotic neurons, Satellitosis, Congested cerebral vasculature and Hemorrhages. Each lesion was scored as follows: 0 = absent, 1 = mild, 2 = moderate, and 3 = severe, based on previously published protocols for assessing neurodegenerative damage^37^.

### Immuno-histochemical technique

Immunohistochemistry (IHC) was performed to evaluate autophagy activity by assessing Beclin-1expression as a key initiator of autophagy and P62 as a reliable marker of autophagy dysfunction^38^. IHC was used to stain the paraffin sections of the hippocampus and cerebral cortex of rats from the various study groups in accordance with^39^ and the manufacturing protocol using anti-Beclin-1 and anti-P62 antibodies (Cambridge, UK, Abcam). All experimental groups’ tissue sections were hydrated and dewaxed. The "DAB chromogenic agent" (Expose mouse and rabbit specific HRP/DAB detection kit, Abcam; Ready-to-use; Cat. #: ab80436) were then used for staining. Hematoxylin counterstaining was then carried out^40^.

A Swift microscope associated with Swift digital camera was used to take all of the pictures of the tissue slices stained by IHC. Five typical areas, comprising both positive cell areas and areas devoid of expression, were chosen for quantitative investigation. Both regions were chosen as representative regions and included in the analysis if a tissue segment had both regions with low and high abundances of labeled cells. J-image analysis software was used to determine the area percentage of immunological positive staining^36^.

### RNA extraction and real-time RT-qPCR

Following manufacturer guidelines, the dissected brain tissue (designated for molecular analysis) was immediately immersed in RNAlater® and stored at 4 °C for 24 h, then transferred to –80 °C until RNA extraction. Total RNA was then extracted using TRIzol™ Reagent** (**Invitrogen, Carlsbad, CA), and quantified using a NanoDrop spectrophotometer. The quality of RNA was confirmed by measuring the A260/A280 ratio: 1.8–2.0. Using the SuperScript™ III First-Strand Synthesis System (Invitrogen), cDNA was synthesized from total RNA. Quantitative real-time PCR was performed using the Platinum™ SYBR™ Green qPCR SuperMix-UDG in the DNA Engine with Chromo4 Detector (MJ Research, Waltham, MA)^41^. RT-qPCR was carried out using the Platinum™ SYBR™ Green qPCR SuperMix-UDG (Invitrogen) in a DNA Engine with Chromo4 Detector (MJ Research, Waltham, MA, USA). Each reaction was performed in a 20 µL total volume, consisting of 10 µL SYBR Green SuperMix, 1 µL forward primer (10 µM), 1 µL reverse primer (10 µM), 2 µL cDNA template, and 6 µL nuclease-free water.

Table 1 lists the primers used in this investigation to identify the mRNA expression of beclin-1, light chain 3 (LC3), and autophagy-related 5 (ATG5).

**Table 1:** Primers sequences for the RT-PCR estimation.

Gene	Primer sequence		
Beclin-1	F:	5′-GCCTCTGAAACTGGACACG-3′	^42^
	R:	5′-CCTCTTCCTCCTGGCTCTCT-3′	
P62	F	5′-TCCCTGTCAAGCAGTATCC-3′	^43^
	R	5′-TCCTCCTTGGCTTTGTCTC-3′	
LC3II	F:	5′-GACTTCCGGAAAGCTCTGCT-3′	^43^
	R:	5′-ACCAGCATCGTAGAGGGTCT-3′	
ATG5	F:	5′-CACTGGGACTTCTGCTCCTG-3′	^42^
	R:	5′-TTCTTCAACCAAGCCAAACC-3-	

### Statistical analysis

The Graph Pad Prism version 5 application was used to assess the data gathered for this study. The Shapiro–Wilk test was employed to ascertain whether the data distribution was normal. The mean ± standard deviation (SD) was used to characterize normally distributed data. To find significant pairs, the Tukey post-hoc test was employed. Due to the heterogeneous distribution of the data, the Kruskal–Wallis Test was utilized to analyze the variations in the semiquantitative way of histopathological scoring. Subsequently, Dunn’s Multiple Comparison test was employed to identify significant pairs.

Additionally, to validate the reproducibility of the histological grading, liver congestion was scored on a 4-point scale (0–3) by two independent observers blinded to the experimental groups. Inter-rater reliability was determined by calculating the percent agreement, Weighted Cohen’s Kappa (quadratic weights), and the Intraclass Correlation Coefficient (ICC). A two-way random-effects model (ICC 2,1) was used to assess the absolute agreement between observers. Statistical analyses were performed using Python (v3.10) with the scikit-learn and statsmodels libraries."

## Results

### Effect of IF on body mass and brain morphology

**Fig. 2:**
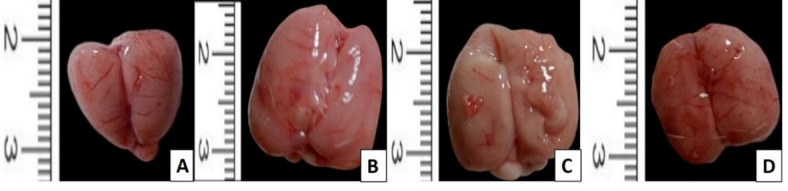
Representative photo-macrograph of rat brain (**A**–**D**) showing: normal size, smooth surface, and regular edges with smooth consistency of brain at control group (A), no macro change appeared at lean fasting group (B), obese group (C), and fasting obese group (D). (*n* = 6).

In the present study, HFD significantly increased final body weight and BMI when compared to the control (p < 0.001 and p < 0.01; respectively) and FL (p < 0.001) groups. In addition, fasting reduced final body weight and BMI in the FO group when compared to the obese non-fasting group (p < 0.001), but both final body weight and BMI in obese, fasting, and non-fasted groups were significantly higher than those of FL group (p < 0.001, p < 0.01). In addition, final body weight and BMI were significantly lower in the FL group in comparison to the control group (p < 0.001 and p < 0.05; respectively) (Table 2). Additionally, no significant changes regarding brain macromorphology were detected among different study groups (Fig. 2).

**Table 2:** Anthropometric measures in all groups.

	Control	Lean fasting	Obese	Fasting obese
Initial body weight (g)	239.2 ± 16.02	241.2 ± 13.14	244.2 ± 12.32	242.5 ± 15.73
Final body weight (g)	359.2 ± 14.29	311.8 ± 20.45^a^	414.5 ± 11.04^ab^	363.2 ± 12.98^bc^
Final BMI (g/cm^2^)	0.51 ± 0.08	0.48 ± 0.02^a^	0.72 ± 0.10^a,b^	0.54 ± 0.05^a,b,c^

Data are expressed as mean ± SD (*n* = 6), ^a^Significant vs control group, ^b^Significant vs Fasting lean group, ^c^Significant vs obese group.

### Effect of IF on social motivation, social memory, and novelty

**Fig. 3:**
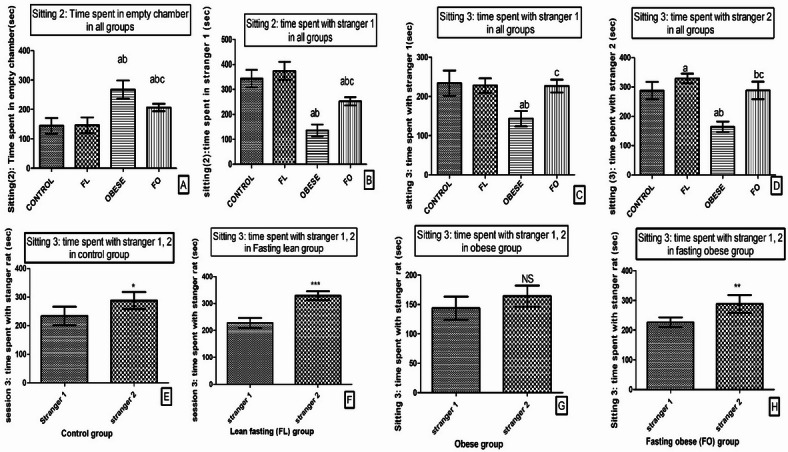
Effect of intermittent fasting on social anxiety behavioral test. (**A**) Session 2: Time spent in the empty chamber, (**B**) Session 2: Time spent with Stranger 1 (chamber 1), (**C**) Session 3: Time spent with Stranger 1 (chamber 1), (**D**) Session 3: Time spent with Stranger 2 (chamber 2), (**E**) Session 3: Time spent with Stranger 1 vs. Stranger 2 in the control group, (**F**) Session 3: Time spent with Stranger 1 vs. Stranger 2 in the lean fasting (FL) group, (**G**) Session 3: Time spent with Stranger 1 vs. Stranger 2 in the obese group, (H) Session 3: Time spent with Stranger 1 vs. Stranger 2 in the fasting obese (FO) group. Data are expressed as mean ± SD (*n* = 6), a: sig vs control group, b: sig vs FL group, c: sig vs obese group. *: p < 0.05, **: p < 0.01, ***: p < 0.001.

Results of Crawley’s Sociability Test indicated that HFD significantly impaired social motivation as indicated by increased time spent in the empty chamber in expense to time spent in social interaction with the stranger rat (1) (135.8 ± 24) which was significantly decreased in comparison to the control group (343.3 ± 35.02) and FL group (FL) (374.5 ± 35.97) (p < 0.001). However, IF in the FO group significantly reversed these changes; time spent in the empty chamber (206.5 ± 12.63 vs 267.3 ± 31.10) was significantly lower (p < 0.01), while time spent in social interaction with the stranger rat (1) (252.2 ± 16.73) was significantly higher than that of the obese non-fasting group (p < 0.001) (Fig. 3A, B).

When comparing the results of this session in the fasting group (FO) with that of control and FL, the time spent in the empty chamber (267.3 ± 31.10, 144.2 ± 26.35 and 146.2 ± 26.75; respectively) was significantly higher (p < 0.01), and the time spent in social interaction with the stranger rat (1) was significantly lower (p < 0.001). However, no significant change was detected in this session between control and FL (p > 0.05) (Fig. 3A, B).

Moreover, in the third setting of the test HFD significantly impaired social novelty as indicated by decreased time spent with strangers 1 (143.5 ± 19.73) when compared to FL (287.8 ± 29.59) (p < 0.001) and (329.0 ± 16.40) (p < 0.001), while fasting in the FO group significantly improved this social novelty impairments; as there was a significant increase in time spent with stranger 1, and 2 (226.5 ± 16.27and 288.3 ± 29.94; respectively) when compared to obese group (p < 0.01 and p < 0.001; respectively) (Fig. 3C, D).

In addition, time spent with strangers 1 showed insignificant change between the control (233.8 ± 32.28) and FL group (p > 0.05) (Fig. 3C, D). Interestingly, time spent with stranger 2 in session 3 was significantly high in the FL group when compared to the control (227.8 ± 18.48) group (p < 0.05). However, this time was insignificantly changed when comparing the FO group with the control group (p > 0.05) (Fig. 3C, D).

**Fig. 4:**
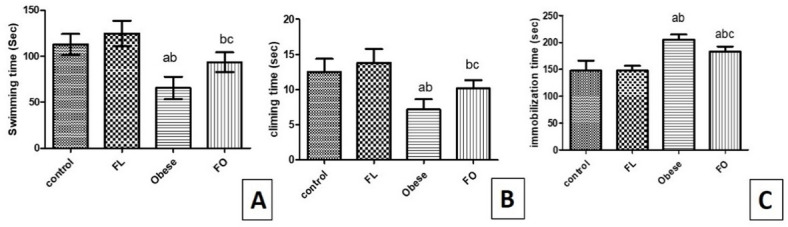
Histogram of Effect of IF on Depressive-like Behavior showing: (**A**) swimming time (s), (**B**) climbing time (s), and (**C**) immobilization time (s) in all groups. Data presented as mean SD (*n* = 6). FL: fasting lean group, FO: fasting obese group. a: significant vs control group, b: significant vs fasting lean group, c: significant vs obese group.

In addition, the time spent with stranger 2 during session 3 was significantly higher than that spent with stranger 1 in the control group (p < 0.05) (Fig. 4E), FL (p < 0.001) (Fig. 3F), and FO (p < 0.01) groups (Fig. 3H). While no significant change was found in the obese group (p > 0.05) (Fig. 3G).

### Effect of IF on depressive-like behavior

The present study showed a significant decrease in swimming time and climbing time associated with an increase in the immobilization time in the obese group (205.5 ± 9.35) in comparison to the control (147.5 ± 18.91) (p < 0.001) and FL (147.8 ± 8.84) group (p < 0.001). However, FO group showed a significant increase in the swimming time (93.50 ± 10.65) (p < 0.01), and climbing time (10.17 ± 1.17) (p < 0.05), with a decrease in immobilization time (183.0 ± 9.59) in comparison to non-fasted rats in the obese group (p < 0.05). While, swimming time and climbing time were significantly lower in the FO than the FL (124.7 ± 13.88, 13.83 ± 1.94; respectively) group (p < 0.01, p < 0.01), with no significant difference in comparison to the control group (112.8 ± 11.2, 12.50 ± 1.87; respectively) (p > 0.05), but immobilization time was significantly higher than those of control group (p < 0.001) and FL group (p < 0.001). When comparing the control to the FL group, no significant difference was detected (p > 0.05) among all previously mentioned test observations (Fig. 4A–C).

### Effect of IF on memory

**Fig. 5:**
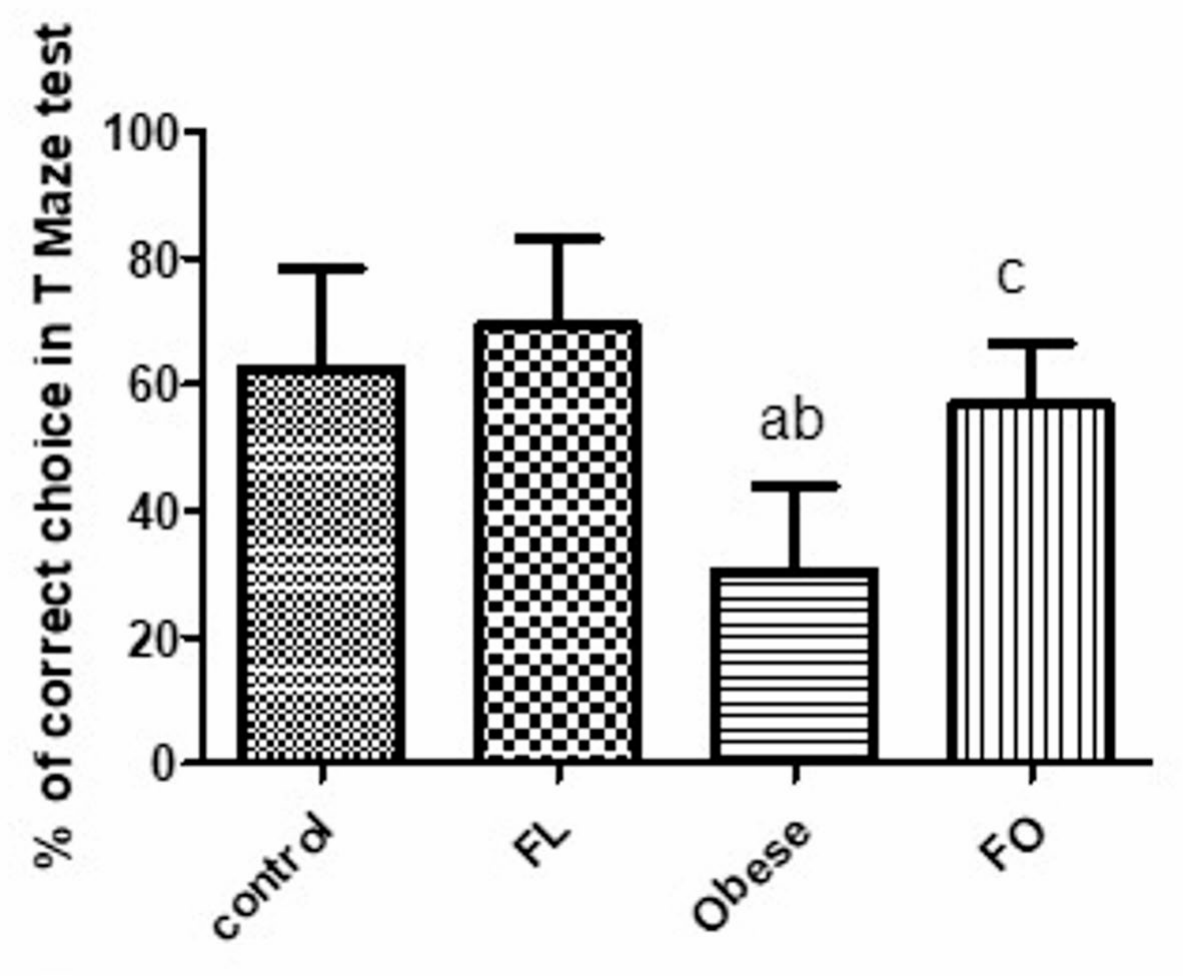
Histogram of Effect of IF on Memory testing by T-Maze test in all groups. Data presented as mean SD (*n* = 6). FL: fasting lean group, FO: fasting obese group. a: significant vs control group, b: significant vs fasting lean group, c: significant vs obese group.

As shown in Fig. 5, the results of the present study showed a significant decrease in % of correct choice done by rats in obese group (30.56 ± 13.61) in comparison to control (62.50 ± 15.59) (p < 0.01) and FL group (69.44 ± 13.61) (p < 0.001). However, fasting reversed this alteration in the FO group (56.94 ± 9.74) when compared to obese non-fasted animals (p < 0.05), which was nonsignificant when compared to the percentage of correct choices in both the control and FL group (p > 0.05). However, no significant change was detected between the control and FL group (p > 0.05) (Fig. 5).

### Effect of IF on inflammatory and oxidative stress markers

The present study showed that HFD significantly increased levels of TNF-α and IL-1B in both serum (p < 0.001) and brain homogenate (p < 0.01 and p < 0.001; respectively) when compared to the control group and also in comparison to FL group (p < 0.001). Interestingly, the FO group exhibited a significant decrease in the serum TNF-α and IL-1B (p < 0.001) and brain homogenate (p < 0.01) when compared to obese non-fasted animals. However, serum TNF-α and IL-1B in the fasting obese group were significantly higher than those of the control group and FL group (p < 0.01). Moreover, brain levels of IL-1B in the FO group were significantly higher than those of control (p < 0.05) and FL group (p < 0.01). Regarding brain TNF-α level, its level showed insignificant change among control, FL, and FO groups (p > 0.05) (Table 3).

**Table 3:** Effect of IF on serum and brain biochemical parameters.

	Control	Fasting lean	Obese	Fasting obese
Serum TNF-α (pg/ml)	58.16 ± 3.5	57.16 ± 4.1	77.1 ± 3.7^a,b^	67.5 ± 1.8^a,b,c^
Serum IL-1B (pg/ml)	22.5 ± 1.23	21.8 ± 0.95	52.16 ± 5.7^a,b^	41.3 ± 5.01^a,b,c^
TNF-α (ng/g brain)	25.8 ± 2.6	21.8 ± 6.6	44.3 ± 9.2^ab^	27.3 ± 7.1^c^
IL-1B (ng/g brain)	5.7 ± 2.2	3.5 ± 1.8	26.0 ± 7.9^ab^	13.8 ± 4.6^abc^
MDA (ng/g brain)	7.9 ± 1.8	3.5 ± 1.3^a^	13.3 ± 1.4^ab^	8.0 ± 1.3^abc^
SOD (U/g brain)	51.2 ± 10.1	61.2 ± 17	25.2 ± 5.7^ab^	42.8 ± 6.4^bc^
BDNF (ng/g brain)	5.3 ± 1.9	8.7 ± 2.2^a^	1.9 ± 0.67^ab^	4.7 ± 1.4^bc^

Data are expressed as mean ± SD (*n* = 6), ^a^Significant vs control group, ^b^Significant vs Fasting lean group, ^c^Significant vs obese group. TNF: Tumor necrosis factor, IL: interleukin, MDA: Malondialdehyde, SOD: superoxide dismutase, BDNF: Brain-derived neurotropic factor.

About oxidative stress markers, MDA showed a significant increase in the obese group in comparison to the control and FL group (P < 0.001), which was associated with a significant reduction in brain SOD (p < 0.01 and p < 0.001; respectively). In fasting obese rats, MDA was significantly reduced, while, SOD was significantly increased in brain homogenate when compared to the obese group (p < 0.001 and p < 0.05; respectively). Moreover, MDA was significantly higher in the FO group when compared to both the control and FL group (p < 0.001), however, SOD was significantly lower in the FO group when compared to the FL group (p < 0.05), with no significant change in comparison to control group (p > 0.05). Brain MDA was significantly low in the FL group when compared to control group (P < 0.001), with no significant difference between both groups as regard to SOD activity (Table 3).

### Effect of IF on brain BDNF

Brain BDNF levels in the current work showed a significant decrease in obese non-fasted animals when compared to control (p < 0.05) and FL (p < 0.001) groups. However, in the FO group, BDNF was higher than that of the obese group (P < 0.05). While, its levels were low in the FO group when compared to the FL group (P < 0.01), with no significant difference when compared to the control group (P > 0.05). While FL group showed a significant increase in BDNF when compared to the control group (P < 0.01) (Table 3).

### Effect of IF on histopathological findings

**Fig. 6:**
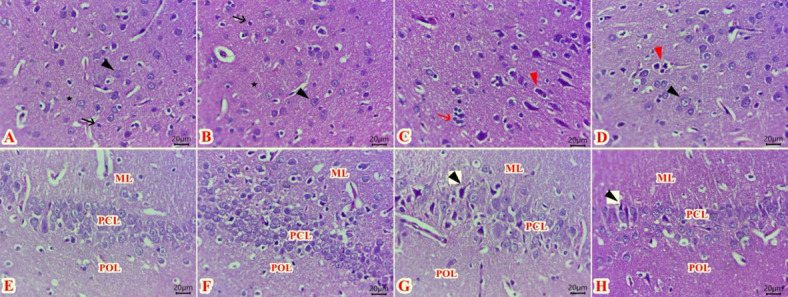
Photomicrographs of H&E-stained sections of cerebral cortex (**A**–**D**) and hippocampus (CA1 region) (E–H) showing: Normal histology of neurons (black arrowheads), glia cells (black arrows), neuropil (stars) and normal vascular tissues at both control (**A**) and fasting lean (**B**) groups. Numerous numbers of pyknotic neurons (red arrowheads), and aggregation of glia cells (red arrow) around some degenerated neurons in obese group (**C**). Few numbers of pyknotic neurons (red arrowhead) and apparent normal most neurons (black arrowhead) in Fasting obese group (**D**). Normal histological structures of polymorphic layer (POL), pyramidal cell layer (PCL) and external molecular layer (ML) in both control (**E**) and fasting lean (**F**) groups. Pyknotic neurons with deeply basophilic nuclei (arrow) at pyramidal cell layer (PCL) beside vacuolated (M) at obese group (**G**). Few numbers of shrunken neuron cell bodies (arrow) at pyramidal cell layer (PCL) at obese fasting group (**H**). (Scale bar 20 μm) (*n* = 6).

Normal histology of neurons, glia cell, neuropil, and vascular tissues were seen in the cerebral cortexes of both control (Fig. 6A) & fasting lean (Fig. 6B). While, sections of the cerebral cortexes of the obese group (Fig. 6C) revealed numerous numbers of pyknotic neurons, and degenerated neurons with aggregation of glia cells around, beside congested cerebral blood vessels with hemorrhage were also encountered. On the other hand, there are ameliorations in histological alterations of the cerebral cortex at the FO group (Fig. 6D) with few numbers of pyknotic neurons. There was a significant increase in Pyknotic neurons, Satellitosis, congested cerebral vasculatures, and hemorrhage scoring in the semiquantitative analysis in the obese group when compared to control and FL animals (p < 0.001) (Table 4).

**Table 4:** The main histopathological lesions score of cerebral cortexes among different experimental groups.

Organ	Main lesions	Control	Fasting lean	Obese	Fasting obese	P value
Cortex	Pyknotic neurons	0	0	3^ab^	1	< 0.0001
	Satellitosis	0	0	2^ab^	1	< 0.0001
	Congested vasculatures	0	0	2^ab^	1	< 0.0001
	hemorrhages	0	0	1^ab^	0^c^	< 0.0001

^a^Significant vs control group, ^b^Significant vs Fasting lean group, ^c^Significant vs obese group.

Regarding hippocampal H&E-stained sections, there was a normal histological structure of the three layers of cornu Ammonias region (CA) of the hippocampus in both control (Fig. 6E), and FL groups (Fig. 6F), which formed from polymorphic layer (POL), pyramidal cell layer (PCL) and external molecular layer (ML). The POL is formed from different glial cells. The PCL separated between POL & ML and comprised from 3 to 5 rows of rounded pyramidal neuronal cell bodies with large rounded open-faced nuclei and a rim of cytoplasm.

The molecular layer (ML) contained branches of apical dendrites of the pyramidal neurons of PCL and randomly distributed small glial cells.

However, numerous numbers of pyknotic neurons with deeply basophilic nuclei at (PCL) beside vacuolated (ML) were seen in the obese group (Fig. 6G). In contrast, few number of shrunken neuron cell bodies surrounded by clear spaces at (PCL) were demonstrated in the FO group (Fig. 6H).

Regarding inter-rater reliability for the histological scoring of cerebral cortex lesion was excellent. The two independent observers achieved 100% absolute agreement across all evaluated samples, yielding a Weighted Cohen’s Kappa of 1.000 and an Intraclass Correlation Coefficient (ICC 2,1) of 1.000 for both Satellitosis and hemorrhage. In addition, they achieved 83.3% absolute agreement across all evaluated samples for Pyknotic neurons and congested vasculature, yielding a Weighted Cohen’s Kappa of 0.944 and 929; respectively and an Intraclass Correlation Coefficient (ICC 2,1) of 0.949 and 0.934; respectively. These findings demonstrate that the semi-quantitative scoring methodology for cortical injury is highly robust and consistently applied (Table 5).

**Table 5:** Inter-rater reliability metrics for histopathological lesions score of cerebral cortexes.

Organ	Main lesions	Percent agreement	Weighted Cohen’s Kappa	Intraclass correlation (ICC)
Cortex	Pyknotic neurons	83.3	0.944	0.949
	Satellitosis	100	1	1
	Congested vasculatures	83.3	0.929	0.934
	hemorrhages	100	1	1

### Effect of IF on immunohistochemical analysis

**Fig. 7:**
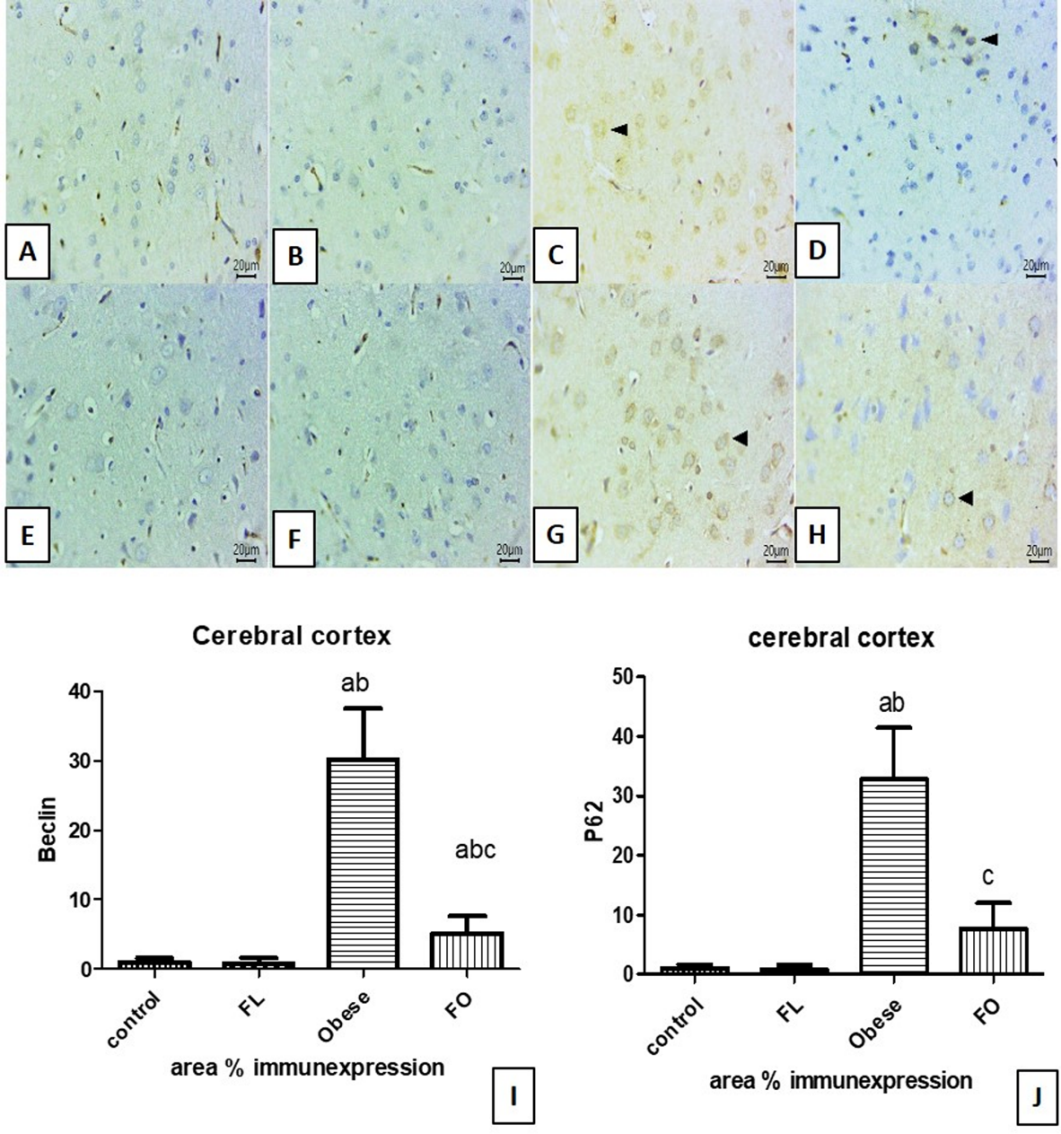
Representative photomicrographs of immune-stained sections from cerebral cortexes for Beclin-1 (**A**–**D**) and p62 (**E**–**H**) of all groups showing: minimal labeling in both control (**A**) and fasting lean groups (**B**). Strong positive cytoplasmic expression for Beclin-1 within abundant numbers of neurons in obese groups (**C**). mild expression of Beclin-1 in the fasting obese group (**D**). Minimal expression in both control (**E**) and fasting lean groups (**F**). Intense positively stained cells in the obese group (**G**). few positive labeled cells in the fasting obese group (**H**). (IHC counterstaining with Mayer’s hematoxylin. Arrowheads refer to positive stained cells. The positive expressed cells revealed a golden-brown color, Scale bar 20μm). Cerebral cortex area % expression of Beclin-1 (**I**) and p62 (**J**). a: significant vs control group, b: significant vs fasting lean (FL) group, c: significant vs obese group, FO: fating obese group. (*n* = 6).

Immunostaining sections from the cerebral cortex against Beclin-1 exhibited minimal expression in both control (Fig. 7A) & fasting lean groups (Fig. 7B). But, the strong positive cytoplasmic expression for Beclin-1 within abundant numbers of neurons was seen in obese group (Fig. 7C). On the other hand, few numbers of immune-stained cells for Beclin-1 were seen in fasting obese group (Fig. 7D) indicating mild expression.

Concerning IHC-stained sections from the cerebral cortex for P62, it showed minimal expression in both control (Fig. 7E) & fasting lean groups (Fig. 7F). While intense numbers of positively stained cells were demonstrated in the obese group (Fig. 7G). Few Positive labeled cells were seen in the FO group (Fig. 7H).

Area % of expression for Beclin-1 and P62 expression in cerebral cortexes within different study groups showed a significant increase in the obese group when compared to the control and fasting lean group (p < 0.001). IF significantly reduced area % expression of both Beclin-1 and P62 in the cerebral cortexes of rats in the fasting obese group when compared to the obese non-fasted group (p < 0.01). While Beclin-1 area % expression was significantly higher than that of control (p < 0.05) and FL group (p < 0.01). On the other hand, no significant difference was noticed among the control, FL, and FO groups (p > 0.05) (Fig. 7I, J).

**Fig. 8:**
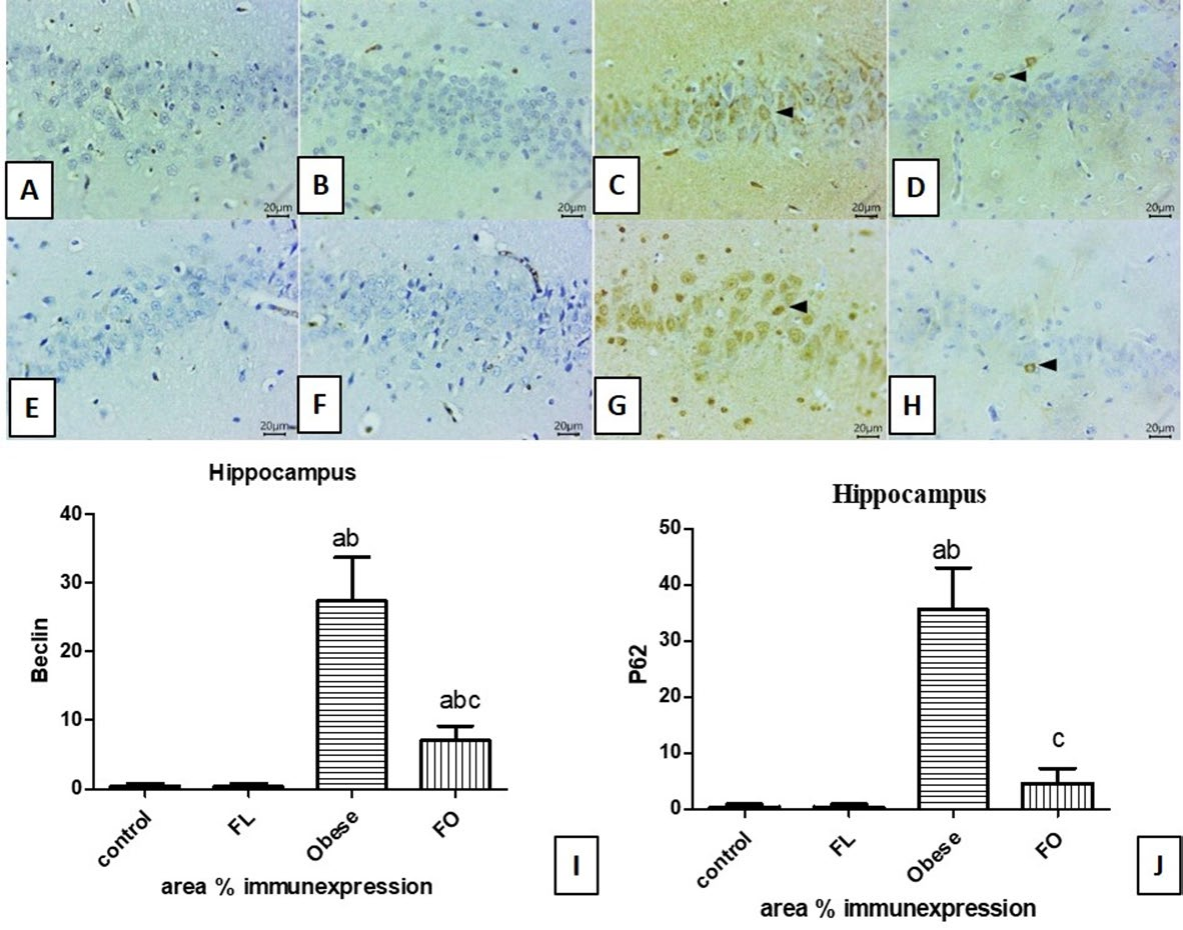
Representative photomicrographs of immune-stained hippocampus sections for Beclin-1 (**A**–**D**) and P62 (**E**–**H**) showing: no immune-expressed cells for Beclin-1 in both control (**A**) and fasting lean groups (**B**). Strong positive cytoplasmic expression within the pyramidal cell layer in the obese group (**C**). Unicellular-staining in obese fasting group (**D**). No expression for P62 in either control (**E**) and fasting lean groups (**F**). Intense positive P62 expression in the obese group (**G**). Few positively labeled cells in the obese fasting group (**H**). (IHC counterstaining with Mayer’s hematoxylin. Arrowheads refer to positive stained cells, the positive expressed cells revealed a golden-brown color. Scale bar 20μm). Hippocampal area % expression of Beclin-1 (**I**) and p62 (**J**). a: significant vs control group, b: significant vs fasting lean (FL) group, c: significant vs obese group, FO: fating obese group. (*n* = 6).

Immunostaining sections from Hippocampus against Beclin-1 exhibited negative expression in pyramidal cell layers of both control (Fig. 8A) & FL groups (Fig. 8B). But, strong positive cytoplasmic expression for Beclin-1 within abundant numbers of neurons were seen at obese group (Fig. 8C). On the other hand, few numbers of immune-stained cells for Beclin-1 were seen in FO group (Fig. 8D) indicating mild expression.

Concerning IHC-stained sections from Hippocampus for P62, it showed negative expression in both controls (Fig. 8E) & fasting lean groups (Fig. 8F). While intense numbers of positive stained cells were demonstrated in the obese group (Fig. 8G). Few Positively labeled cells were seen in the FO group (Fig. 8H).

Area % of expression for Beclin-1 and P62 expression in the hippocampus within different study groups showed a significant increase in the obese group when compared to the control and fasting lean group (p < 0.001). IF significantly reduced area % expression of both Beclin-1 and P62 in the cerebral cortexes of rats in the fasting obese group when compared to the obese non-fasted group (p < 0.01). Beclin-1 area % expression was significantly higher than that of control (p < 0.05) and FL group (p < 0.05). On the other hand, no significant difference was noticed among the control, FL, and FO groups (p > 0.05) (Fig. 8I, J).

### Effect of IF on autophagy proteins mRNA expression

The current work showed a significant increase in Beclin-1–1 and P62 mRNA expression in the obese group when compared to control (p < 0.01, p < 0.001; respectively) and FL group (p < 0.01, p < 0.001; respectively), while, in FO group Beclin-1 and P62 mRNA expression were significantly lower than that of obese non-fasted animals (p < 0.05, p < 0.001; respectively). However, only P62 expression was significantly higher than that of the FL group (P < 0.01). No significant difference was observed between the control and FL group (P > 0.05), or between the control and FO group (P > 0.05) (Table 6).

**Table 6:** Autophagy proteins mRNA expression.

	Control	Fasting lean	Obese	Fasting obese
Beclin-1	0.07 ± 0.02	0.035 ± 0.02	0.34 ± 0.26^ab^	0.1 ± 0.02^c^
P62	1.1 ± 0.2	0.55 ± 0.2	2.98 ± 0.7^ab^	1.7 ± 0.4^bc^
LC3-II	0.15 ± 0.05	0.29 ± 0.07^a^	0.04 ± 0.02^ab^	0.15 ± 0.04^bc^
ATG5	0.17 ± 0.05	0.35 ± 0.13^a^	0.035 ± 0.02^ab^	0.16 ± 0.04^bc^

Data are expressed as mean ± SD (*n* = 6), ^a^Sig vs control group, ^b^Sig vs Fasting lean group, ^c^Sig vs obese group.

Regarding LC3-II and ATG5, the present work showed a significant decrease in LC3-II and ATG5 mRNA expression in the obese group when compared to control (p < 0.01, p < 0.05; respectively) and FL group (p < 0.001). However, their mRNA expression was significantly increased in the FO group when compared to obese non-fasted animals (P < 0.01, p < 0.05; respectively). Moreover, in the FL group, both LC3-II and ATG5 expression were significantly higher than that of control (p < 0.01) and FO groups (p < 0.01) (Table 6).

## Discussion

High-fat diet (HFD)–induced obesity is considered a major global health concern. This lifestyle pattern is characterized by excessive caloric intake and reduced energy expenditure, which increase the risk of developing chronic diseases such as ischemic stroke, atherosclerosis, and type 2 diabetes^41,42^. In addition, HFD has been associated with the induction of neuroinflammation, which is closely linked to impaired cognitive performance, including memory deficits^43^. Similar findings were observed in the present study.

When compared to the animals in the control group fed a regular diet, we discovered that HFD consumption resulted in a reduction in cognitive performance. The present study assessed three aspects of cognitive functions; first was the anxiety behavior that was investigated using Crawley’s sociability test which showed a significant decrease in time spent in stranger chambers in all sessions in the obese group indicating impaired social motivation and the insignificant change between time spent with stranger 1 and 2 in sitting 3 of this group indicated impaired social memory and novelty^44^. The second test was depressive-like behavior that was investigated using a forced swimming test, the results showed a significant decrease in swimming time and climbing time associated with an increase in the immobilization time in this group when compared to the control one. The third aspect was a modified T-maze test that was used to assess memory performance, and it showed a significant decrease in % of right choices taken by the obese animals of this group in comparison to the control group.

Similar to previous research, rats fed a high-fat diet (HFD) showed a decline in cognitive function^3^ and rats with type 2 diabetes also showed this effect^45^. Similar to this, HFD aggravates behavioral abnormalities in a variety of animal models of anxiety-like behaviors^46^, which may be caused by oxidative stress and inflammation damaging the brain^47^, However, it is still unclear how an HFD impairs memory, which depends on a healthy hippocampus.

As previously reported, HFD disrupts the cholinergic system by upregulating acetylcholinesterase expression, leading to accelerated breakdown of acetylcholine. This disruption contributes to oxidative stress and memory impairment, and subsequently stimulates calcium (Ca^2+^) influx–mediated release of pro-oxidant amyloid-beta peptides^48^.

Interestingly in the present study, HFD administration for 8 weeks significantly produced a state of systemic and local brain inflammation and oxidative stress as proved by a significant increase in serum and brain inflammatory mediators; IL-1B, and TNF- α and oxidative stress marker MDA, in addition to, neuroinflammation in brain cerebral cortex and in the synapto-somal fraction from the brain cortex of the HFD mice linked to increase in TNF-α secretion^47^.

Furthermore, they connected mitochondrial dysfunction to this inflammatory condition. Mitochondria, which are found at synapses and play a critical role in supplying energy to support synaptic functions and plasticity—both of which are critical for intellectual functions—play a critical role in regulating energy homeostasis in the central nervous system (CNS). Defects in these mitochondria may result in synaptic failure, which is the cause of neurodegenerative diseases^49^. Interestingly, the cellular and molecular mechanisms by which IF improves health and counteracts disease processes involve activation of adaptive cellular stress response signaling pathways that enhance mitochondrial health, DNA repair and autophagy ^16^ which is consistent with reduction of MDA in fasting lean group.

The cognitive protection observed with IF intervention in HFD-fed rats recommended a beneficial impact on HFD-induced metabolic disruption via the reduction of inflammatory and oxidative stress processes, which was accompanied by decreased histological alterations as pyknotic neurons, satellitosis, and hemorrhage in H&E analyzed sections. As in previous results, IF improved HFD-induced memory deficits by reducing neuroinflammation and inhibiting blood–brain barrier breakdown^50^. Furthermore, IF diminishes inflammation by a decrease in blood monocytes^51^.

Meanwhile, BDNF signaling in the brain is impacted by neuroinflammation^52^. Similar to previous findings, the HFD brain homogenate showed a marked reduction in BDNF expression^53^. As evidenced by lower levels of mature-BDNF in the bloodstream and a lower mature-BDNF/pro-BDNF ratio in people with depression and bipolar disorder, BDNF is a neurotrophins that is crucial for the CNS’s plasticity and also plays a role in the etiology of neurological disorders^54,55^. BDNF specifically affects synaptic plasticity in the near term by causing post-translational modifications to proteins that are already present at the synapse, but it also has long-term impacts, such as modifying the synaptic system of protein production^56^, triggering neuronal growth and distinction, cell survival, long-term potentiation, and synaptic plasticity. Fasting acts as a metabolic stressor that triggers adaptive neuronal signaling, which increased BDNF in fasting lean group when compared to control group. This observation can be explained by the effect of Ketone bodies (β-hydroxybutyrate) produced during fasting that activate cAMP/PKA/p-CREB signaling pathways that promotes BDNF transcription in the hippocampus ^58.^

Moreover, Liu et al. ^58^ demonstrated that treadmill exercise exerts anti-neurodegenerative effects in early Alzheimer’s disease models by modulating the miR-34a/TAN1/CREB signaling axis. Their findings showed that exercise enhanced BDNF expression, promoted oligodendrocyte trophic support, and facilitated myelin repair, while simultaneously attenuating astrocyte-mediated neuroinflammation. These results support the role of BDNF as a key neuroprotective factor, not only in inhibiting neuronal apoptosis and promoting remyelination, but also in counteracting neuroinflammation. In line with these findings, the reduction of BDNF observed in our HFD group may partly explain the associated cognitive deficits, whereas the protective effect of intermittent fasting could be mediated through restoration of BDNF signaling.

The brain uses ketones like β-hydroxybutyrate and acetoacetate as its preferred fuel during fasting. These ketones are transferred from the liver to the brain, where they are converted back to acetyl CoA and HMG-CoA, which causes BDNF to be upregulated^57^. In addition, Fasting was found to inhibit the mTOR pathway leading to an improvement in antioxidant defenses, DNA repair, and stimulation of BDNF^58^, leading to the promotion of mitochondrial biogenesis, synaptic plasticity, and cellular stress resistance in animal models^59^ and human^60^. Also, it was theorized that greater circulating BDNF during IF leads to an increase in BDNF in the brain^59^, however, in the present study we measured BDNF in the brain only.

Despite cognitive dysfunction following HFD feeding being poorly understood, impaired autophagy, a process where neurons remove dysfunctional or damaged components, is involved in lysosomal storage disorders, and neurodegenerative diseases, such as AD^61,62^.

Because it may start the creation of autophagosomes, mediate the recruitment of autophagy-related proteins (ATG), and encourage the production and maturation of autophagosomes, Beclin-1 is an essential molecule in autophagy^63^. Furthermore, light chain 3β (LC3), a microtubule-associated macro-autophagy protein that is necessary for autophagosome production and fusion with lysosome. Lipidated LC3-II and soluble LC3-I are the two kinds of LC3. ATG first activates LC3I, and one of the most important processes in autolysosomes is the transformation of LC3 from the LC3-I-cleaved form to the conjugated form (LC3II)^64^.

An adverse haemodynamic profile and diminished blood flow were observed in adipose tissue and cerebral cortex from obese cases, suggesting a hypoxic state in the tissue, which likely contributes to metabolic dysfunction and increased autophagic flux, that later results in impaired autophagy-mediated clearance^65^. Emerging evidence suggests direct links between impaired autophagy and Aβ accumulation^66^. Normally, Aβ aggregates can also be degraded by the autophagy-lysosomal pathway (ALP), so defective ALP is characterized by increased formation of autophagosomes and Aβ_40_ and Aβ_42_ accumulation^67^. In addition, ablation of Atg5 or Atg7 in neurons results in spontaneous neurodegeneration^68^.

Remarkably, in this study by increasing Beclin-1, P62 immune and mRNA expression within both the cerebral cortex and hippocampus, which was associated with decreased LC3II and ATG5 mRNA in the obese group. As in previous findings, there are increased P62 and decreased LC3I, II, and Atg in the hepatic tissues of HFD-fed mice^69^.

However, fasting obese rats showed a reverse of these parameters when compared to obese non-fasted animals. The reduction of Beclin-1 in fasting was in line with other previous research as Beclin-1expression after one month of fasting even in healthy individuals was important to avoid unnecessary apoptosis in healthy tissues without decreasing the basal autophagy process^58^. Moreover, earlier results that IF protected against age-induced benign prostatic hyperplasia in rat models via anti-inflammatory and antiproliferative effects, suppression of oxidative stress, and by improving autophagy via Beclin-1/P62 modulation^70^. In addition, IF improved both cerebellar changes prompted by HFD through reestablishing the autophagy balance^71^.

Beclin-1 may promote autophagy early on, but since autophagy is followed by lysosome fusion and content destruction, this does not mean that HFD-fed mice have a better autophagic system. Similar results showed that the heart tissues of HFD-fed mice had more autophagosomes but fewer autolysosomes, suggesting that autophagosome formation was normal or even increased in HFD-fed mice when compared to normal control mice. However, the autophagic flux was suppressed in HFD-fed mice^72^.

Notably, in the same line, fasting for two weeks augmented Beclin-1 expression level and activated autophagy, however, after 1 month of fasting Beclin-1 was reduced, indicating the dynamical and more complex function of Beclin-1in autophagy^73^. Similarly, autophagy can also be induced independently from Beclin-1 and categorized as a non-canonical autophagy pathway^74^. 

According to earlier research, P62 contributes to aggregation formation and builds up in reaction to molecular aggregates and the advancement of disease^75^, which could be explained by a deficit in the clearance of pathological accumulation ^76^, and an indicator of autophagy inhibition, and deficits in downstream autophagosomal pathways^77^.

Prior research revealed elevated cytoplasmic p62 expression in dementia patients’ cerebrospinal fluid^78^, and also its high levels were associated with aggressive tumor behavior and poorer prognosis^77^. Thus, high LC3/ low p62 may be indicative of activated and intact autophagy, and low LC3/p62 may show low basal autophagy^79^. Autophagy was upregulated in cerebral cortex neurons in rats with vascular cognitive impairment and was associated with learning and memory impairment, that was reversed by down-regulating the level of autophagy in cerebral cortex neurons^80^. Previous studies found that lifestyle enhancement, including diets regimens to prevent the metabolic syndrome of obesity are linked to a lowered the hazard for memory defect, and autophagy failure is strongly supposed to have an imperative role in promoting this syndrome^81^.

The relevance of the autophagy system to normal brain activities was highlighted by the current investigation, which showed that IF had a preventive effect against HFD-induced cognitive impairments that may be mediated by cerebral cortex and hippocampus autophagy dysfunction. Future research will be able to identify obese patients with poor cognitive disorders by analyzing the complex relationship between Beclin-1, p62, and LC3 expression. It would also be very interesting to apply these analyses to female animals to look into any potential gender differences in the brain’s reaction to HFD and IF.

This study has some limitations. First, the small sample size (n = 6) may restrict the statistical power and limit the generalizability of the results. Second, the investigation was conducted using a single intermittent fasting model (three alternate days per week, 24-h fasting), which may not capture the potential variability in response to different fasting protocols. Third, while both mRNA expression and immunohistochemical localization of autophagy-related proteins were evaluated, the study did not include quantitative protein-level analysis which could have provided more precise data on protein expression levels. Also, the reliance on static measurements of autophagy-related markers as we measured only LC3II mRNA expression, which do not fully capture the dynamic nature of autophagic flux. Since the LC3II/LC3I protein ratio provides a more accurate assessment, future studies should include both LC3II/LC3I analysis and autophagy flux assays using lysosomal inhibitors to better characterize autophagic activity. Additionally, long-term effects of intermittent fasting were not assessed, and the findings reflect short-term molecular and histological changes only.

## Conclusions

IF had a preventive effect against HFD-induced cognitive disorders that could be mediated by the cerebral cortex and hippocampal autophagy dysfunction, emphasizing the importance of the autophagy pathway to normal neuronal functions. These results suggested that IF protected the neural system from HFD-induced inflammation and oxidative stress in obese rats and is essential for neuronal survival via modulation of autophagy function in rats.

However, future studies should Incorporate quantitative protein analysis is recommended to validate transcriptional and histological findings. Additionally, longitudinal studies evaluating both the short- and long-term effects of intermittent fasting on autophagy, tissue integrity, and functional outcomes would provide deeper insights.

## Data Availability

Data will be made available on request.
